# Equid herpesvirus 8: Complete genome sequence and association with abortion in mares

**DOI:** 10.1371/journal.pone.0192301

**Published:** 2018-02-07

**Authors:** Marie Garvey, Nicolás M. Suárez, Karen Kerr, Ralph Hector, Laura Moloney-Quinn, Sean Arkins, Andrew J. Davison, Ann Cullinane

**Affiliations:** 1 Virology Unit, The Irish Equine Centre, Johnstown, Naas, County Kildare, Ireland; 2 MRC-University of Glasgow Centre for Virus Research, Glasgow, United Kingdom; 3 Department of Life Sciences, University of Limerick, Limerick, Ireland; University of Melbourne, AUSTRALIA

## Abstract

Equid herpesvirus 8 (EHV-8), formerly known as asinine herpesvirus 3, is an alphaherpesvirus that is closely related to equid herpesviruses 1 and 9 (EHV-1 and EHV-9). The pathogenesis of EHV-8 is relatively little studied and to date has only been associated with respiratory disease in donkeys in Australia and horses in China. A single EHV-8 genome sequence has been generated for strain Wh in China, but is apparently incomplete and contains frameshifts in two genes. In this study, the complete genome sequences of four EHV-8 strains isolated in Ireland between 2003 and 2015 were determined by Illumina sequencing. Two of these strains were isolated from cases of abortion in horses, and were misdiagnosed initially as EHV-1, and two were isolated from donkeys, one with neurological disease. The four genome sequences are very similar to each other, exhibiting greater than 98.4% nucleotide identity, and their phylogenetic clustering together demonstrated that genomic diversity is not dependent on the host. Comparative genomic analysis revealed 24 of the 76 predicted protein sequences are completely conserved among the Irish EHV-8 strains. Evolutionary comparisons indicate that EHV-8 is phylogenetically closer to EHV-9 than it is to EHV-1. In summary, the first complete genome sequences of EHV-8 isolates from two host species over a twelve year period are reported. The current study suggests that EHV-8 can cause abortion in horses. The potential threat of EHV-8 to the horse industry and the possibility that donkeys may act as reservoirs of infection warrant further investigation.

## Introduction

Nine herpesviruses have been identified in the family Equidae, which includes horses, ponies, donkeys and zebras. It is understood that the horse (*Equus ferus caballus*) is the natural host of five of these viruses (equid herpesvirus (EHV) 1 (EHV-1), EHV-2, EHV-3, EHV-4 and EHV-5), and the donkey (*Equus africanus asinus*) the natural host of three (EHV-6, EHV-7 and EHV-8, the last formerly known as asinine herpesvirus 3 (AHV-3)) [1, 2]. It has been suggested that the natural host for the ninth virus (EHV-9), which was isolated originally from gazelle [3], is the zebra, but serological prevalence points to the African rhinoceros as at least an additional potential natural host [4]. The EHVs are classified into species *Equid alphaherpesvirus 1* to *Equid alphaherpesvirus 9*, and five (EHV-1, EHV-3, EHV-4, EHV-8 and EHV-9) belong to genus *Varicellovirus* in subfamily *Alphaherpesvirinae* of the family *Herpesviridae* [5]. Alphaherpesviruses are characterized by lytic infection, and can establish a lifelong latent infection that may be interrupted by periodic reactivation [6]. EHV-1 is globally ubiquitous, and is considered the most economically significant EHV because it is associated with abortion and neurological disease, including myeloencephalopathy [7].

AHV-3 was first isolated in 1987 from the nasal cavity of donkeys in Australia following experimental administration of high doses of corticosteroid [2, 8]. Based on sequence analysis of gene ORF70 (encoding glycoprotein G) and serological cross reactivity of AHV-3 antibodies with EHV-1 and EHV-4 glycoproteins, AHV-3 was categorised as an alphaherpesvirus [9] and subsequently designated EHV-8 [5, 10]. In 2010, the ostensibly complete genome sequence of EHV-8 strain Wh, which was isolated from horses in China, was published [11]. The sequences of gene ORF30 (encoding the DNA polymerase catalytic subunit) and the partial sequence of gene ORF70 of the Australian donkey strain 804/87 [9] are the only EHV-8 data additional to strain Wh that are available in GenBank.

The pathogenesis of EHV-8 is not well understood, and to date the virus has been associated only with respiratory disease. On experimental intranasal infection of naïve weanling donkeys, strain 804/87 induced afebrile rhinitis [9]. EHV-8 strain Wh was isolated from horses with fever and nasal discharge. The present study contains the first report of EHV-8-associated abortion in horses. In addition, the complete genome sequences of four EHV-8 strains isolated in Ireland between 2003 and 2015 are presented, two originating from horses and two from donkeys, with one of the latter from a neurological case.

## Materials and methods

### Viral isolation and identification by PCR

The tissues of two horse foetuses aborted in the third trimester were diagnosed by pathological examination as originating from EHV-associated abortions. Post mortem tissues were received by the Virology Unit for identification of the causal agent as EHV-1 or EHV-4. The abortions occurred in 2003 and 2010 in two different counties in Ireland (Co. Kerry and Co. Kildare). Viral DNA was initially detected in both samples by PCR using primers specific for EHV-1 ORF16 (encoding glycoprotein C) [12]. The viruses were isolated from tissue homogenates by a single passage in rabbit kidney (RK13) cell monolayers [13]. EHV-4 and equine arteritis virus were not detected in the tissue samples by PCR.

PCR of a sequence of approximately 500 bp in EHV-1 ORF30 that contains a putative neurological marker [14] in the DNA polymerase catalytic subunit was carried out by using primers 30.2141.F (5’-TGGTTGTGTTTGACTTCGCT-3’) and 30.2655.R (5’-GTAGATAACCCTGACGGAGTA-3’). The sequence of the PCR product showed a high level of similarity to EHV-1 for both isolates. However, BLAST analysis [15] carried out at this stage (prior to deposition into GenBank in 2012 of the genome sequence of EHV-8 strain Wh; accession no. JQ343919 [11]) showed that the closest relative was EHV-9 rather than EHV-1. To enable comparison with EHV-8, PCR products were generated and sequenced by using primers AHV3.gG. 915.F (5’-CTTACGGAGACATCAACG-3’) and AHV3.gG.1200.R (5’-GCCTGAGCCAAGATTCT-3’), which target a 286 bp region of ORF70 in the only EHV-8 sequence available at the time [9] (from an Australian strain; GenBank accession U24184, 1585 bp).

In addition to these two isolates from horses, which were designated EHV-8/IR/2003/19 and EHV-8/IR/2010/47, viruses were obtained from two nasal swabs taken from donkeys in Co. Cork, one of which was exhibiting respiratory signs and the other neurological signs. These samples were submitted for virus detection in 2010 and 2015, and AHV-3 was detected with the AHV-3 ORF70 primers. The viruses were then isolated by a single passage in RK13 cell monolayers. These isolates were designated EHV-8/IR/2010/16 and EHV-8/IR/2015/40, respectively.

The characterisation of archived isolates from diagnostic specimens was approved by the Board of Governors of the Irish Equine Centre.

### ORF30 sequencing

The subsequent availability of the EHV-8 strain Wh genome sequence and of the ORF30 sequence from EHV-8 strain 804/87 [16] allowed sets of overlapping primers to be designed by using Primer3 [17], in order to facilitate the amplification and sequencing of the whole of ORF30. Viral DNA from the four viruses was isolated on first passage from the RK13 cell culture supernatant by using a QIAamp DNA mini kit (QIAGEN, Hilden, Germany). The amplified PCR products were purified by using a QIAquick gel extraction kit (QIAGEN) and sequenced commercially (QIAGEN Genomic Services, Hilden, Germany). The sequences were assembled by using BioEdit sequence alignment editor version 7.2.5 [18] and aligned by using ClustalW [19].

### Genome sequencing

Each virus was passaged a further three times in RK13 cells. DNA was then extracted from infected cells by using a Hi-Pure PCR purification kit (Roche Diagnostics, Indianapolis, IN, USA) and quantified by using a Nanodrop 1000 spectrophotometer (Thermo Scientific, Wilmington, DE, USA).

Sequencing libraries were prepared from 5 μg DNA as described previously [20], and sequenced on a NextSeq instrument (Illumina, San Diego, CA, USA) to generate paired-end reads of 150 nucleotides (nt). The numbers of reads trimmed of adapters and low quality data are listed in Table 1. The reads for EHV-8/IR/2003/19 and EHV-8/IR/2010/47 were assembled *de novo* by using SPAdes version 3.5.0 [21], and the resulting contigs were ordered against the sequence of EHV-8 strain Wh in order to produce draft genome sequences. The draft genome sequences of EHV-8/IR/2010/16 and EHV-8/IR/2015/40 were produced by mapping the read data to the draft EHV-8/IR/2003/19 genome by reference-based assembly using Bowtie 2 version 2.2.6 [22]. Alignments of the respective data sets against the four draft genome sequences were visualised by using Tablet version 1.14.11.07 [23], and the consensus sequences were corrected manually. No major variants were noted in any of the sequences. The sizes of 16 tandem reiterations in each genome were determined by generating eight PCR products and sequencing them using internal primers (Sigma-Aldrich). The genome termini were mapped by identifying sets of sequence reads that shared a common end in the regions corresponding to the EHV-1 and EHV-4 genome termini [24–26]. Locating the genome termini led to the construction of the complete genome sequences. The numbers of reads mapping to each sequence, the coverage depths and the GenBank accessions are listed in Table 1.

**Table 1:** Summary of sequencing and genome data for the four Irish EHV-8 strains.

	EHV-8/IR/2003/19	EHV-8/IR/2010/47	EHV-8/IR/2010/16	EHV-8/IR/2015/40
Total trimmed reads (no.)	3,281,110	4,231,272	3,624,440	2,810,830
EHV-8 reads (no.)	237,890	1,207,021	886,413	175,652
Coverage depth (reads/nt)	237	1195	878	175
Genome size (bp)	149,600	149,800	149,405	149,667
U_L_ size (bp)	112,966	113,368	112,917	113,337
U_S_ size (bp)	12,148	12,316	12,358	12,346
TR_L_/IR_L_ size (bp)	32	32	32	32
TR_S_/IR_S_ size (bp)	12,211	12,026	12,033	11,960
GenBank accession no.	MF431611	MF431612	MF431613	MF431614
European Nucleotide Archive study accession no.	PRJEB24126	PRJEB24126	PRJEB24126	PRJEB24126

Abbreviations: EHV-8, equid herpesvirus 8, U_L_, unique long region; U_S_, unique short region; TR_L_/IR_L_ and TR_S_/IR_S_, terminal and internal inverted repeats flanking U_L_ and U_S_, respectively.

Protein-coding regions were assigned on the basis of the EHV-1 model [24]. Comparative analysis of predicted amino acid sequences was carried out by using ClustalW implemented in Bioedit. The genomes of EHV-1 strain V592 (N752 strain, GenBank accession AY464052), EHV-1 hypervirulent strain AB4 (D752 strain, GenBank accession AY665713) and EHV-9 strain P19 (GenBank accession AP010838) were included in these comparisons. The corresponding amino acid sequences for each strain were generated as Fasta files, pairs of sequences were aligned for each protein, and pairwise identity values were calculated by using the BLOSUM62 similarity matrix. Pairwise identity range values were calculated for each alignment from the minimal and maximal identity values of the four EHV-8 strains in comparison with EHV-1 and EHV-9.

### Phylogenetic analysis

An alignment containing the four Irish EHV-8 genome sequences and that of EHV-8 strain Wh was created by using Gap4 [27], and used as the basis for comparison. Multiple sequence alignments of the predicted amino acid sequences of individual EHV-8 genes and of related genomes (S1 Table) were created by using Clustal implemented in Bioedit. Phylogenetic trees were computed by implementing the maximum likelihood method [28] in MEGA7 version 7.0.14 [29]. The optimal model for each tree was chosen on the basis of the lowest Bayesian information criterion (BIC) score. Further investigations of phylogenetic relationships were carried out by aligning the concatenated amino acid sequences of 74 of the 76 viral genes, implementing the JTT matrix-based model [30] with 1000 bootstrap replicates. ORF1 and ORF2 were omitted from this analysis because the genome of EHV-1 strain T529 10/84 lacks the 1,611 bp region containing these coding regions [31]. EHV-8 strain Wh was also omitted because of the presence of frameshifted genes.

## Results

### Detection of EHV-8

Prior to the availability of the EHV-8 strain Wh genome sequence, analysis of the 287 bp portion of ORF70 of the two viruses from horse foetuses revealed differences of only 1–2 nt in comparison with the Australian EHV-8 strain. The two isolates from donkeys were identical to EHV-8/IR/2003/19 in ORF70. These results identified the four Irish viruses as being isolates of EHV-8. The levels of identity of the corresponding regions in the closest related genomes (those of EHV-1 and EHV-9) were approximately 95% (i.e. differences of up to 15 nt).

### ORF30 sequences

Comparison of the nucleotide sequences of ORF30 for the four Irish strains showed that they share 99.67–99.97% identity. The sequences obtained through Sanger sequencing using overlapping PCR products were identical to those obtained during the whole genome/Ilumina read assembly. Horse strain EHV-8/IR/2003/19 is more closely related to asinine strain EHV-8/IR/2010/16 than to horse strain EHV-8/IR/2010/47. The levels of identity to EHV-8 strains Wh and 804/87 are 99.5–99.7% and 99.5–99.6%, respectively. The four Irish strains are slightly more closely related to EHV-9 (approximately 95%) than they are to EHV-1 (approximately 93%).

**Fig 1:**
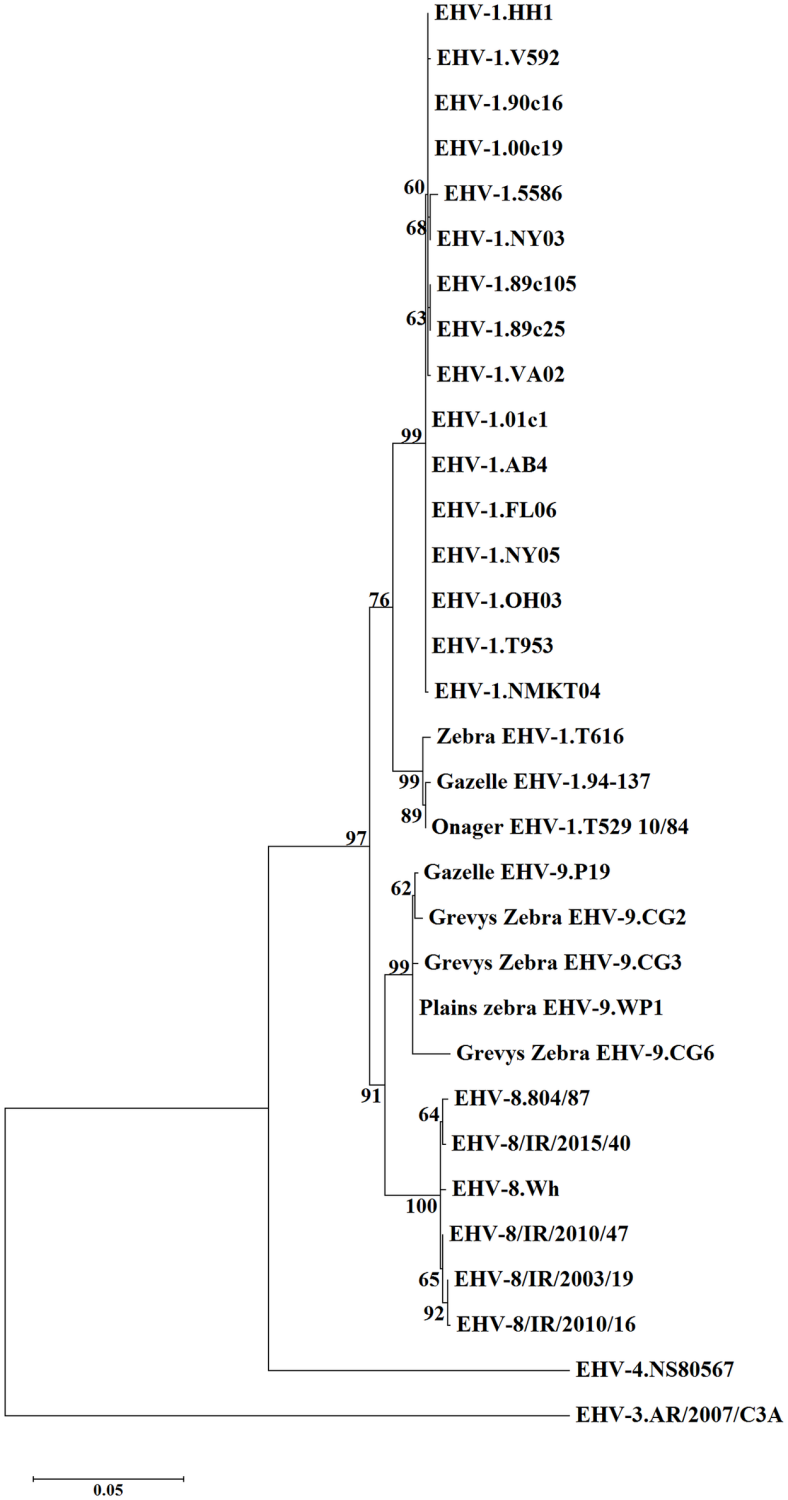
Maximum likelihood phylogenetic tree of ORF30 amino acid sequences based on the JTT matrix-based model. Details of the sequences are in S1 Table of the Supporting Information. The scale bar represents the number of substitutions per site. Bootstrap values after 1000 replications are indicated at major nodes.

At the level of predicted amino acid sequences, the four Irish strains are 99.51–99.92% identical to each other, 99.51–99.75% identical to EHV-8 strain Wh and 99.34–99.67% identical to EHV-8 strain 804/87. All EHV-8 strains have an aspartic acid (D) residue at position 752 in the ORF30 protein, which corresponds to the putative D752 hypervirulence marker (formerly identified as a marker of neuropathogenicity) in EHV-1. The level of identity to EHV-9 strain P19 is approximately 97%, and those to EHV-1 strains AB4 and V592 are slightly lower, at 96% and 95%, respectively. To illustrate the relationship between the viruses, a phylogenetic tree was constructed (Fig 1). The four new Irish strains, EHV-8 strain Wh and EHV-8 strain 804/87 form a separate clade that is more closely related to EHV-9 than EHV-1.

### Genome sequences

**Fig 2:**
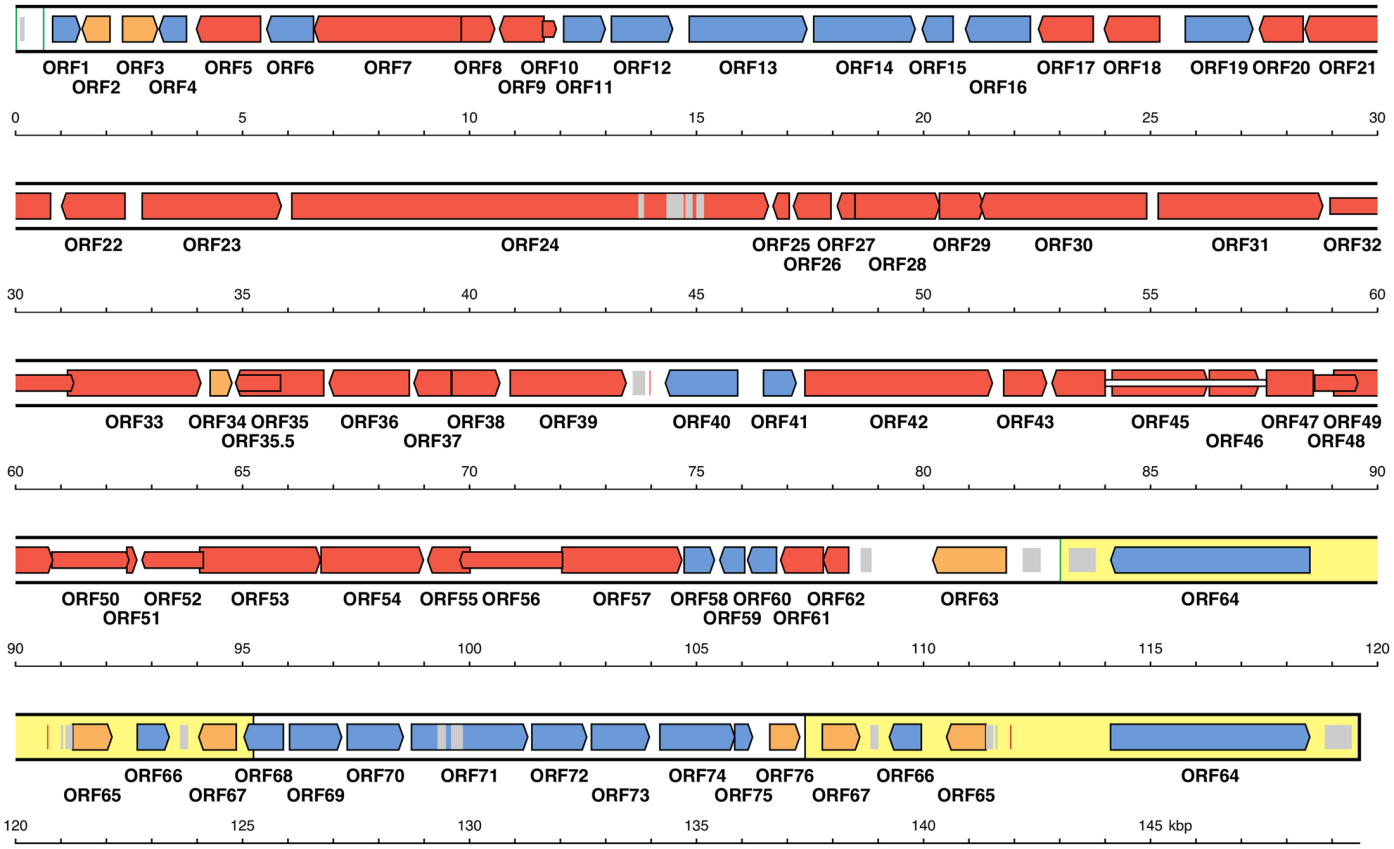
Map of the equid herpesvirus 8 (EHV-8) genome. The coordinates are based on the 149600 bp sequence of EHV-8/IR/2003/19. Predicted protein-coding regions are shown by coloured arrows with names below. Red open reading frames (ORFs) were inherited from the ancestor of family *Herpesviridae* and blue ORFs from the ancestor of subfamily *Alphaherpesvirinae*. Orange ORFs are specific to certain lineages within subfamily *Alphaherpesvirinae*. Inverted repeats TR_S_/IR_S_ flanking U_S_ are coloured yellow, and inverted repeats TR_L_/IR_L_ flanking U_L_ are shown by green vertical lines at 1–32 and 112999–113030 bp. A third green vertical line marks a third copy of the TR_L_/IR_L_ sequence that is part of a more extensive inverted repeat at 1–87 and 547–633 bp. Tandem reiterations are marked by grey shading, and origins of DNA replication by vertical red lines.

The linear genome of EHV-8 consists of unique long and short regions (U_L_ and U_S_) flanked by inverted terminal and internal repeats (TR_L_/IR_L_ and TR_S_/IR_S_) in an arrangement (TR_L_-U_L_-IR_L_-IR_S_-U_S_-TR_S_) that is typical of other varicelloviruses, including EHV-1 and EHV-9. The genome has the same complement and arrangement of 80 open reading frames (ORFs) predicted to encode functional proteins as EHV-1 and EHV-9, of which 76 are unique and four are duplicated in TR_S_/IR_S_ (Fig 2). The size of the four Irish EHV-8 genomes ranges from 149405 to 149800 bp (Table 1). The component sizes are: U_L_ (113 kbp), U_S_ (12 kbp), TR_L_/IR_L_ (32 bp) and IR_S_/TR_S_ (12 kbp). The average nucleotide composition of the whole genome is 54.5–54.6% G+C, and that of IR_S_/TR_S_ is substantially higher (64.61–64.75% G+C).

The four Irish EHV-8 genome sequences are 98.42–99.03% identical to each other. EHV-8/IR/2003/19 and EHV-8/IR/2010/16, which were isolated from a horse and donkey, respectively, share the highest level of identity to each other (99.03%; 1461 substitutions and indels), and the two viruses isolated from aborted horse foetuses (EHV-8/IR/2003/19 and EHV-8/IR/2010/47) share the lowest (98.42%; 2377 substitutions and indels). Levels of identity to EHV-8 strain Wh are lower (approximately 97%) for all four strains, and those to EHV-9 and EHV-1 are lower still, at approximately 92% and 89%, respectively. Many of the differences between the Irish strains and EHV-8 strain Wh are located in ORF64 (in TR_S_/IR_S_) and ORF71 (in U_S_).

**Fig 3:**
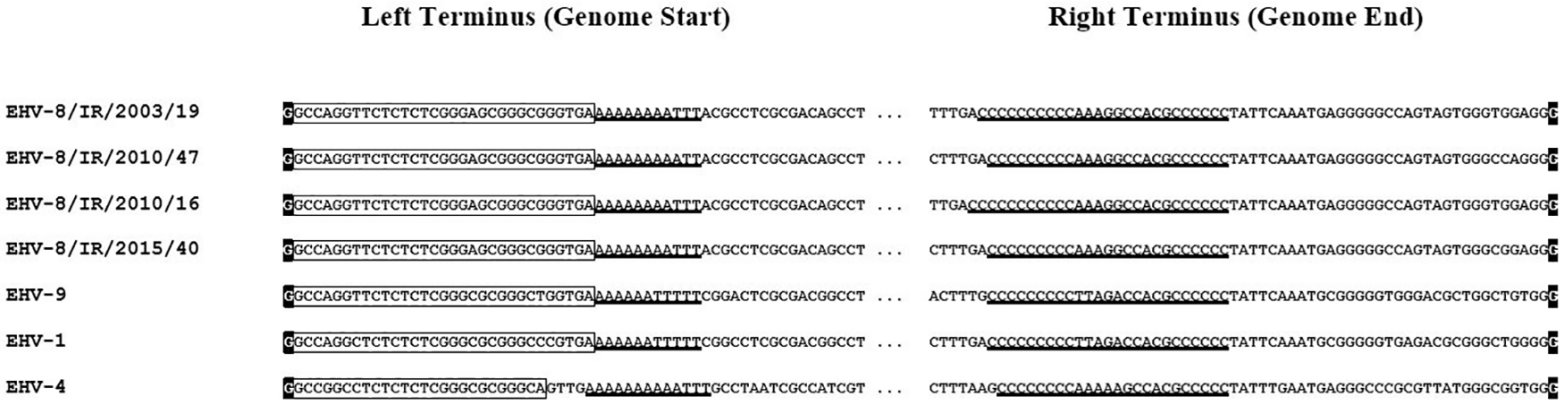
Conserved sequences near the genome termini of equid varicelloviruses. An alignment of sequences from the four Irish equid herpesvirus (EHV) 8 (EHV-8) strains, EHV-9 P19 (AP010838.1), EHV-1 AB4 (AY665713.1) and EHV-4 NS80567 (AF030027.1) is shown. In each sequence, TR_L_ is boxed, the terminal nucleotides are shaded black, and conserved regions AnTn and γ (which are present near the left and right termini, respectively) are underlined. Ellipses denote the remainder of the genome.

Alignment of sequences near the termini of the four Irish EHV-8 genomes with those of other varicelloviruses demonstrated the conservation of sequence elements (AnTn and γ) [32] that are required for the maturation of unit-length genomes from replicated concatemers (Fig 3). Compared with the Irish EHV-8 genomes, the EHV-8 strain Wh genome lacks a region of 398–601 bp at the left terminus and has an extra region of 1304 bp at the right terminus that corresponds to the region at the right end of U_L_.

### Tandem reiterations

Sixteen tandem reiterations consisting of multiple copies of short, G+C-rich sequences (plus a partial copy) were identified in the genomes of the sequenced EHV-8 strains. Five are duplicated in TR_S_/IR_S_ and seven form parts of protein-coding regions (four in ORF24 and three in ORF71) (Table 2). The number of repeat units differs among isolates, and three reiterations exhibit minor sequence variations in the unit sequence. EHV-8 strain Wh lacks four of the reiterations, three being part of ORF71 in the Irish strains and the other being located in the region near the left genome terminus that is not represented in the strain Wh sequence. The number of repeat units in some reiterations was substantially fewer in EHV-8 strain Wh than in the Irish strains.

**Table 2:** Tandem reiterations in EHV-8 genomes.

					Number of units				
Location (bp)^1^	Protein-coding region	Unit size (bp)	Partial unit size (bp)	Unit sequence (5’-3’)	EHV-8/IR/2003/19	EHV-8/IR/2010/47	EHV-8/IR/2010/16	EHV-8/IR/2015/40	EHV-8 Wh
97–199	/ ^2^	17	5	CACCGGGAGGCCACGCC	6	18 (1+17^3^)	9 (3+6^3^)	13	_^4^
43719–43743	ORF24	63	–^4^	TCCTTCCACCCTTCCACCCGCTTCCCCTCTACC CCAACCCCCTTCAAAAGCCGCCAGCGGCAC	2	4	2	5	5
44338–44718	ORF24	33	18	GCCGCGGCCCCGGCCAAATCTGCGGCGGCCCCC	11	18	10	6	1
44754–44915	ORF24	12	6	GGCCAAGGACCA	13	9	11	14	7
44995–45165	ORF24	45	36	GAAAACACTCCCCTACCAGATGACTCGCCCATTG GAGCTGTCCCC	3	3	2	3	2
73593–73868	/	32	20	GCGGGAGCGAGGGCTGCTGCGGCGGCGGTGCG	8	9	7	12	1
108616–108855	/	18	6	TAGCGCTAACGCTAGGGC	13	12	21	28	11
112183–112582	/	14	7	TGCCATAGTCCCCC	28	16	20	15	15
113199–113499 (149134–149432) ^5^	/	15	_^4^	GGAAGGGGAGGAGCA	20	13 (6+7^3^)	19	19	14
113500–13795 (148836–149131) ^5^	/	12	_^4^	CCATCAACCCGC	24	18	8	11	12
120998–121049 (141582–141633) ^5^	/	14	10	GTGGTCGGTGGTCG	3	10 ^3^	5	6	11
121096–121257 (141374–141535) ^5^	/	20	2	CCCAGCTCCAGCGACCCCAG	8	2	4	5	2
1213625–123802 (138829–139006) ^5^	/	22	2	CAGAGCTGGGAGCCGAGTGGGG	8	9	12	6	8
129291–129481	ORF71	15	11	GCGGCTACCACCACA	12	17	18	17	_^4^
129594–129632	ORF71	15	7	ACCACATCTACCCCT	2	9	3	5	_^4^
129631–129855	ORF71	15	–^4^	CTGAGACATCAACAG	15	13	22	19	_^4^

Abbreviations: EHV-8, equid herpesvirus 8; bp, base pair; ORF, open reading frame.

^1^ In EHV-8/IR/2003/19.

^2^ Not located in a predicted protein coding region.

^3^ Repeat units exhibiting sequence variation.

^4^ Not present.

^5^ Inverted sequence in TR_S_ (terminal inverted repeat flanking U_S_ (unique short sequence)).

### Origins of DNA replication

Three potential origins of DNA replication were identified in the Irish EHV-8 genomes, each being partially palindromic and containing inverted copies of a diagnostic 9 bp sequence (CGTTCGCAC) separated by an A+T-rich sequence [33]. OriS is located in IR_S_/TR_S_ between ORF64 and ORF65, and OriL is located in the centre of U_L_ between ORF39 and ORF40 (Fig 2).

### Conservation of protein-coding regions among EHV-8 strains

The predicted amino acid sequences of 73 of the 76 predicted protein-coding regions are ≥99% identical among the Irish strains, the exceptions being ORF24, ORF64 and ORF71. The numbers of predicted amino acid differences in relation to EHV-8/IR/2003/19 (excluding regions encoded by tandem reiterations) are summarised in S2 Table. A total of 24 ORFs are completely conserved among the Irish strains, and 16 of these are also completely conserved in EHV-8 strain Wh. Among the Irish strains, the largest number of amino acid differences from EHV-8/IR/2003/19 was recorded for EHV-8/IR/2010/47 (87 differences across 32 proteins). Strain EHV-8/IR/2015/40, isolated from a donkey with neurological signs, has 86 amino acid differences from EHV-8/IR/2003/19 in 42 proteins.

The level of identity among the Irish strains in ORF24 (encoding the large tegument protein) is 95.61–99.03%. These lower values are largely due to differences in the lengths of tandem reiterations (3499–3602 residues). Likewise, the levels of identity between the Irish strains and EHV-8 strain Wh is lower (93.65–97.75%) because the latter has shorter tandem reiterations in ORF24 (3445 residues).

The level of identity among the Irish strains in ORF64 (encoding transcriptional regulator ICP4) is 97.08–99.93%. EHV-8/IR/2010/47 is the most divergent, having 33 differences from EHV-8/IR/2003/19, with the majority of these located at the N terminus. Isolate EHV-8/IR/2015/40 has 14 differences from EHV-8/IR/2003/19, with all but one of these being common to EHV-8 strain Wh. However, the EHV-8 strain Wh protein has a much lower overall level of identity (87.80–90.58%) to the Irish EHV-8 proteins. This is due to a frameshift in the EHV-8 strain Wh gene that results in a C-terminally truncated protein. This truncation leads to the absence of conserved amino acid sequence motifs that are required for gene regulation in other alphaherpesviruses [34].

ORF71 (encoding gp2, which is equivalent to herpes simplex virus type 1 (HSV-1) glycoprotein J) is the least conserved of the 76 predicted proteins, having a level of identity among the Irish strains of 92.42–97.60%. Most differences are due to three tandem reiterations that differ in length among strains. Strain Wh shares only 62.10–69.60% identity to the Irish strains, due in part to the absence of two of the reiterations that are common to the Irish strains and in part to a frameshift-induced truncation of the strain Wh protein to 544 residues, compared to 853–923 residues in the Irish strains.

### Conservation of EHV-9 and EHV-1 protein-coding regions in EHV-8

Pairwise sequence alignments indicate that the level of amino acid sequence divergence between the Irish EHV-8 strains and EHV-1 strain V592 ranges from 73.18 (ORF71) to 99.18% (ORF44) (Table 3), with 34, 28 and 14 proteins exhibiting >95, 90–94 and <90% identity, respectively, to their EHV-1 equivalents. In contrast, the levels of identity between the EHV-8 strains and EHV-9 strain P19 range from 74.89 (ORF71) to 99.45% (ORF44), with 46, 21 and 9 proteins sharing >95, 90–94 and <90% identity, respectively, to the corresponding EHV-9 proteins (Table 3). In addition, the majority of ORFs (74 out of 76) share a higher level of identity to EHV-9 strain P19 than EHV-1 V592, the exceptions being ORF41 and ORF75.

**Table 3:** Amino acid sequence identity of genes of the Irish EHV-8 strains to their homologues in EHV-1 and EHV-9.

ORF	HSV-1 equivalent ^1^	Protein	Identity to EHV-1 strain V592 (range, %)	Identity to EHV-9 strain P19 (range, %)
1	UL56	Membrane protein UL56	91.08	**93.06** ^2^
2	_	Membrane protein V1	85.85–86.34	**88.72–89.21**
3	_	Myristylated tegument protein CIRC	90.27–90.66	**93.77–94.16**
4	UL55	Nuclear protein UL55	92.50	**95.50–95.55**
5	UL54	Multifunctional expression regulator	93.40–93.61	**95.53–95.74**
6	UL53	Envelope glycoprotein K	97.95	97.95
7	UL52	Helicase-primase primase subunit	93.06–93.24	**95.46–95.64**
8	UL51	Tegument protein UL51	95.91–96.32	**97.55–97.95**
9	UL50	Deoxyuridine triphosphatase	96.62–97.23	96.62–97.23
10	UL49A	Envelope glycoprotein N	94.0–95.0	**97.0–98.0**
11	UL49	Tegument protein VP22	91.47	**95.40**
12	UL48	Transactivating tegument protein VP16	92.20–92.43	**93.10–93.31**
13	UL47	Tegument protein VP13/14	84.73–84.95	**85.30–85.55**
14	UL46	Tegument protein VP11/12	91.86–92.0	**95.29–95.43**
15	UL45	Membrane protein UL45	85.38–85.84	**91.63–92.07**
16	UL44	Envelope glycoprotein C	93.06–93.27	**93.48–93.69**
17	UL43	Envelope protein UL43	96.25	**97.75**
18	UL42	DNA polymerase processivity subunit	96.05–96.31	**97.78–98.02**
19	UL41	Tegument host shut-off protein	94.36	**97.58**
20	UL40	Ribonucleotide reductase subunit 2	96.26–96.57	**96.88–97.19**
21	UL39	Ribonucleotide reductase subunit 1	96.32	**97.59**
22	UL38	Capsid triplex subunit 1	95.05–95.26	**98.28–98.49**
23	UL37	Tegument protein UL37	93.43–93.53	**94.22–94.31**
24	UL36	Large tegument protein	86.95–89.66	**88.8–90.55**
25	UL35	Small capsid protein	95.79–96.63	**96.63–97.47**
26	UL34	Nuclear egress membrane protein	94.54–94.90	**97.81–98.18**
27	UL33	DNA packaging protein UL33	88.54–89.31	**93.84–94.61**
28	UL32	DNA packaging protein UL32	89.69–89.85	**94.68–94.85**
29	UL31	Nuclear egress lamina protein	97.85	**98.46**
30	UL30	DNA polymerase catalytic subunit	95.81–96.06	**97.04–97.29**
31	UL29	Single stranded DNA- binding protein	97.35–97.43	**97.76–97.84**
32	UL28	DNA packaging terminase subunit 2	96.64	**96.9**
33	UL27	Envelope glycoprotein B	96.22–96.32	**97.16–97.38**
34	_	Protein V32	87.50–88.12	**90.62–91.25**
35	UL26	Capsid maturation protease	96.59–96.74	**97.36–97.52**
35.5	UL26.5	Capsid scaffold protein	96.35	96.35
36	UL25	DNA packaging tegument protein UL25	95.74–96.08	**96.93–97.27**
37	UL24	Nuclear protein UL24	94.5	**94.74**
38	UL23	Thymidine kinase	97.15	**98.01**
39	UL22	Envelope glycoprotein H	95.65	**96.47**
40	UL21	Tegument protein UL21	96.98	**97.73**
41	UL20	Envelope protein UL20	**97.07**	96.58
42	UL19	Major capsid protein	99.05–99.12	**99.20–99.27**
43	UL18	Capsid triplex subunit 2	97.77	**98.72**
44	UL15	DNA packaging terminase subunit 1	99.04–99.18	**99.32–99.45**
45	UL17	DNA packaging tegument protein UL17	96.17–96.31	**96.74–96.88**
46	UL16	Tegument protein UL16	95.13–95.40	**97.28–97.56**
48	UL14	Tegument protein UL14	90.53	**93.71**
49	UL13	Tegument serine/threonine protein kinase	94.61–94.78	**96.30–96.49**
50	UL12	Deoxyribonuclease	95.04	**96.1**
51	UL11	Myristylated tegument protein	90.54	**94.52**
52	UL10	Envelope glycoprotein M	97.11	**98.22**
53	UL9	DNA replication origin-binding protein	97.51	**98.13**
54	UL8	Helicase-primase subunit	92.27–92.41	**95.87–96.0**
55	UL7	Tegument protein UL7	94.38–95.04	**97.02**
56	UL6	Capsid portal protein	95.21–95.48	**96.81–97.10**
57	UL5	Helicase-primase helicase subunit	96.14–96.36	**96.93–97.16**
58	UL4	Nuclear protein UL4	90.66–91.11	**93.75–94.19**
59	_	Protein V57	82.96–83.51	**90.65–91.20**
60	UL3	Nuclear protein UL3	96.69	**97.64**
61	UL2	Uracil- DNA glycosylase	91.05	**96.11–96.60**
62	UL1	Envelope glycoprotein L	88.24	**89.83**
63	RL2	Ubiquitin E3 ligase ICP0	86.13–86.66	**87.29–87.82**
64	RS1	Transcriptional regulator ICP4	90.37–91.52	**94.37–95.87**
65	US1	Regulatory protein ICP22	94.53–94.88	**96.56–96.90**
66	US10	Virion protein US10	93.22–93.64	**94.44–94.87**
67	_	Virion protein V67	92.7	**94.89**
68	US2	Virion protein US2	84.15–84.48	**89.86–90.54**
69	US3	Serine/threonine protein kinase US3	93.19	**93.73**
70	US4	Envelope glycoprotein G	91.97–92.21	**92.21–92.45**
71	US5	Envelope glycoprotein J	73.18–75.83	**74.89–77.33**
72	US6	Envelope glycoprotein D	90.79–91.04	**91.29**
73	US7	Envelope glycoprotein I	90.56	**92**
74	US8	Envelope glycoprotein E	95.63–95.81	**97.09–97.27**
75	US8A	Membrane protein US8A	**84.61–85.38**	83.84–84.61
76	US9	Membrane protein US9	82.64	**86.30**

Abbreviations: EHV-8, equid herpesvirus 8; EHV-1, equid herpesvirus 1; EHV-9, equid herpesvirus 9; HSV-1, herpes simplex virus type 1; ORF, open reading frame; UL, US, RL and RS, genes located in U_L_, U_S_, TR_L_/IR_L_, TR_S_/IR_S_, respectively (U_L_, unique long region; U_S_, unique short region; TR_L_/IR_L_ and TR_S_/IR_S_, terminal and internal inverted repeats flanking U_L_ and U_S_, respectively); VP, viral protein; ICP, infected cell protein

^1^ Nomenclature of HSV-1 homologues or positional counterparts. Genes lacking HSV-1 positional counterparts are indicated by hyphens.

^2^ The higher identity range in each row is in bold type.

**Fig 4:**
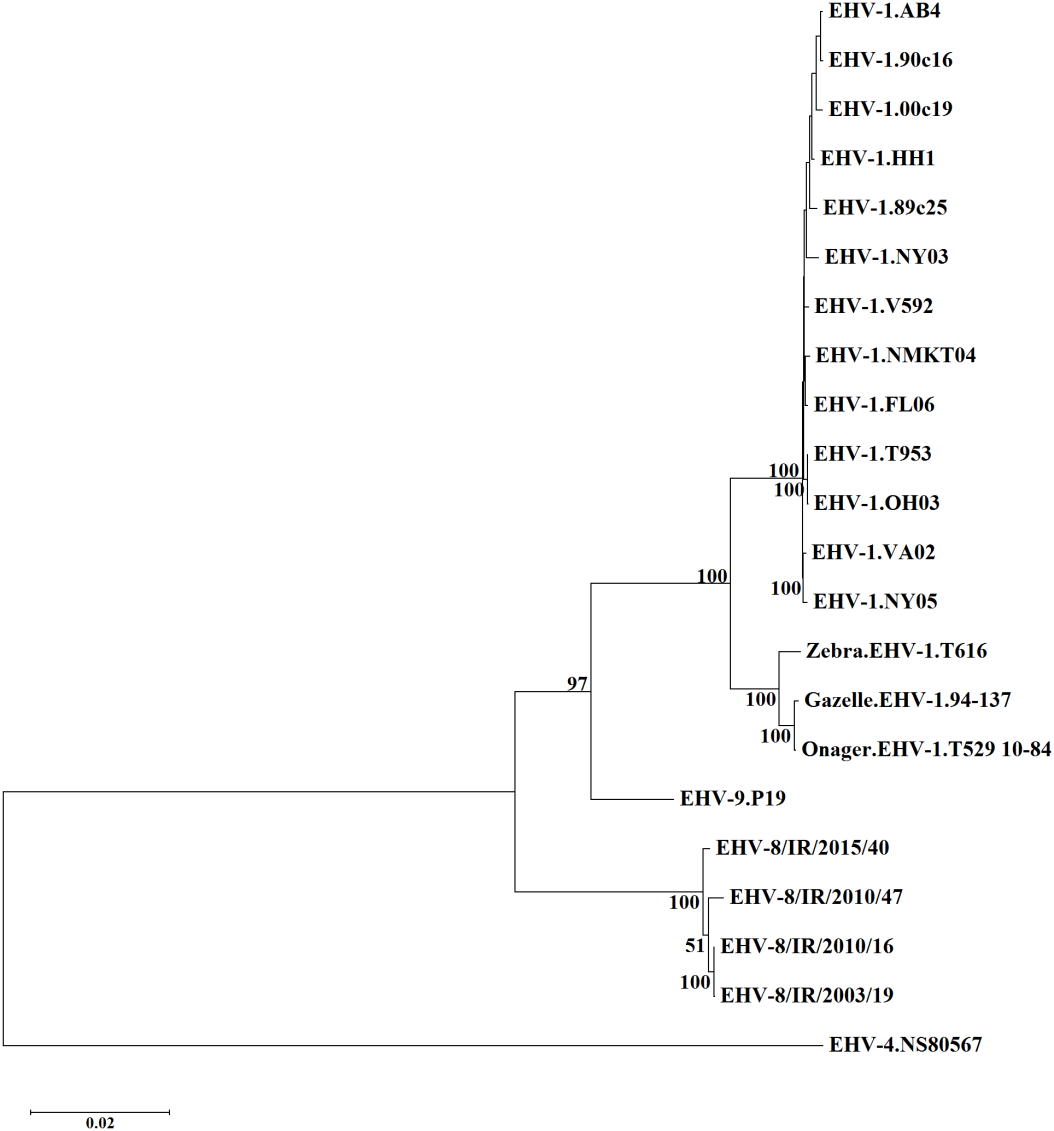
Phylogenetic tree of concatenated amino acid sequences of ORF3-ORF76 from the Irish equid herpesvirus 8 (EHV-8) strains, EHV-8 strain Wh and other equid alphaherpesvirus strains. The tree was constructed by using the maximum likelihood method based on the JTT matrix-based model [30], and is drawn to scale. Bootstrap values from 1000 replicates are shown as percentages at major nodes.

An additional evolutionary comparison was generated by constructing a phylogenetic tree based on a concatenation of the amino acid sequences of 74 ORFs (Fig 4). This demonstrated that the four Irish EHV-8 strains cluster separately from EHV-9 and EHV-1 strains.

## Discussion

The complete genome sequences of four EHV-8 strains isolated in Ireland between 2003 and 2015 were determined. Two of the strains were isolated from the aborted foetuses of horses, which on the basis of gross post mortem findings and histopathological examination had been diagnosed as EHV-associated abortions. A further two strains were isolated from nasal swabs from donkeys, one exhibiting neurological signs and the other respiratory disease. The sequences were determined by Illumina sequencing and compared with that of EHV-8 strain Wh, which was determined by Sanger technology [11], and those of other equid alphaherpesviruses. EHV-8 shares a common genome structure with other equid alphaherpesviruses in the genus *Varicellovirus*, whereas the EHV-8 strain Wh sequence lacks the accurate identification of the genome termini [32] and is apparently incomplete at the left terminus. In addition, frameshifts are apparent in two genes (ORF64 and ORF71) of EHV-8 strain Wh, and at least the former is likely to represent sequencing errors. The sequences of the four Irish strains thus represent the first complete EHV-8 genome sequences to be reported.

Phylogenetic analysis indicates that the Irish EHV-8 strains exhibit minimal diversity. They cluster together and are closely related to, but distinct from, EHV-9 and EHV-1. Sequence alignments revealed a low degree of heterogeneity among strains isolated from two host species over a 12 year period. EHV-8/IR/2003/19 and EHV-8/IR/2010/16 show the highest similarity of the Irish EHV-8 isolates at both the nucleotide and amino acid levels, despite having been isolated from a horse and donkey, respectively. In contrast, EHV-1 strains isolated from gazelle, onager and Grevy’s zebra form a separate genetic group from EHV-1 strains isolated from horses [35].

Although donkeys have been reported to be the natural hosts for EHV-8 [9], our data demonstrate that the virus has the capability to cross host species and cause abortion in horses. Cases of natural infection by EHV-9, which is closely related to EHV-8, have been reported in multiple species including gazelles [3], zebras [36] and giraffes [37], and the virus has been isolated from an aborted Persian onager in a zoo [36]. Infections caused by cross-species transmission of herpesviruses can result in increased virulence and cause severe or fatal diseases. For example, EHV-9 has been associated with meningoencephalitis in a polar bear that necessitated euthanasia [38], and fatal acute encephalitis has been induced by experimental infection in goats [39] and pigs [40]. EHV-9-inoculated horses exhibited mild encephalitis but lacked vasculitis, which is one of the main pathological lesions of EHV-1 neurologic disease [41]. EHV-1 has been isolated from non-equid species including cattle [42], deer [43], antelope [44], alpacas and llamas [45]. Wohlsein et al. [46] identified D752 EHV-1 in Thomson’s gazelles, black bear and guinea pigs in two different zoo epizootics that were associated with abortion, severe neurological signs and high mortality rates.

It is possible that EHV-8 is under-diagnosed in horses because of its close relationship to other equid alphaherpesviruses. Indeed, the EHV-8 strains isolated from two aborted foetuses of horses in this study were identified incorrectly as EHV-1 during initial testing, due to cross reactivity of the PCR assay. Viral causes of abortion in horses have only been attributed to EHV-1, and less frequently EHV-4 and equine viral arteritis [47]. In future, EHV- 8 will need to be included in the differential diagnosis. The availability of EHV-8 genome sequences will facilitate the development of a specific PCR assay for EHV-8 in aborted foetuses and neonatal foal deaths, and will also enable further investigation into the putative of role of EHV-8 in equid neurological disease. In this study, we report the first isolation of EHV-8 from a donkey with neurological disease. The number of outbreaks of neurological disease in horses attributed to the EHV-1 hypervirulent phenotype may be inflated, as may the increased prevalence of this phenotype in EHV-1 abortions [48], due to the inclusion of EHV-8 cases. The ORF30 variation (D752/N752) has been applied to the development of PCR tests to discriminate between EHV-1 hypervirulent and non-hypervirulent strains [49–51]. All sequenced EHV-8 strains have the D752 genotype, and the specificity of the EHV-1 allelic discrimination test has not been assessed for EHV-8 [49]. Given the high level of sequence identity to EHV-8 at the primer-binding sites used in this assay [49], with one mismatch in the forward primer and none in the reverse primer or the probe (G2254), there is a strong likelihood of misidentified detections. Furthermore, an assessment of the EHV-1 gC primers used initially in this study [12] showed that there were only two mismatches in the forward primer and one in the reverse primer.

The EHV-8 strains isolated from the two horse abortions originated in two distinct provinces approximately 7 years apart. The source of infection in either case was unknown. Donkeys and horses occasionally share pasture in Ireland, but neither mare had a history of contact with donkeys during the gestation period. Moreover, cross-species transmission of EHV-1 and EHV-9 has been reported in animals that do not have direct contact. It has been hypothesised that water can act as a source of herpesvirus infections, and EHV-1 has been shown to remain infectious in water for up to three weeks [52]. However, there is currently no evidence to support the survival of EHV-8 in water as a basis for cross-species transmission to horses. As an alternative, the possibility that EHV-8 circulates continuously in horses, even at a low level, merits investigation. One potentially useful corollary to the availability of the EHV-8 genome sequences would be the development of an EHV-8-specific peptide-based ELISA similar to that developed for the detection and differentiation of EHV-1- and EHV-9-specific antibodies [53]. This ELISA could be used to determine the seroprevalence of EHV-8 infections in donkeys and horses. Alternatively, the available sequence information makes it possible to develop PCR assays to investigate, by examination of existing or latent virus populations in horses, whether these viruses represent sporadic events in which the donkey virus has crossed into individual horses, or whether EHV-8 is circulating in some horse populations. This approach has been employed previously when trying to detect co-infections with neuropathogenic versus abortigenic strains [54] or in a wider range of equids [4].

The co-occurrence of EHV-1 and EHV-8 also needs to be monitored because EHVs have the potential to diversify rapidly by recombination. Recombinant EHV-1/EHV-9 infections that originated in asymptomatic zebras were reported to cause non-fatal and fatal encephalitis in polar bears [55] and abortion and neurological disease in Indian rhinoceroses [56].

In conclusion, the current study suggests that EHV-8 can cause abortion in horses. The potential threat of EHV-8 to the horse industry and the possibility that donkeys may act as reservoirs of infection warrant further investigation. The determination of complete genome sequences of EHV-8 strains will serve as a key reference for the development of specific assays for diagnosis and epidemiological research.

## Supporting information

S1 Table GenBank accession numbers of equid alphaherpesvirus sequences used in phylogenetic analyses. (DOCX)

S2 Table Number of amino acid sequence differences among the Irish equid herpesvirus (EHV-8) strains. (DOCX)
