# Supplementary material for: Equid herpesvirus 8: Complete genome sequence and association with abortion in mares
Source: PLoS One. 2018 Feb 7;13(2):e0192301. doi: 10.1371/journal.pone.0192301 (PMC5802896; doi:10.1371/journal.pone.0192301)
Supplement: S1 Table — (DOCX) [file pone.0192301.s001.docx]

# **S1 Table. GenBank accession numbers of equid alphaherpesvirus sequences used in phylogenetic analyses**

| **Source** | **Virus** | **Strain name** | **ORF30 accession^1^** | **Genome accession^2^** |
| --- | --- | --- | --- | --- |
| Horse | EHV-1 | 00c19 | AII81443.1 | KF644576.1 |
| Horse | EHV-1 | 01c1 | [AII81603.1](https://www.ncbi.nlm.nih.gov/protein/671708006) | [AII81644.1](https://www.ncbi.nlm.nih.gov/protein/671708047) |
| Horse | EHV-1 | 5586 | BAM75880.1 | AP012321.1 |
| Horse | EHV-1 | 89c105 | [AII81523.1](https://www.ncbi.nlm.nih.gov/protein/671707925) | [AII81564.1](https://www.ncbi.nlm.nih.gov/protein/671707966) |
| Horse | EHV-1 | 89c25 | AII81683.1 | KF644579.1 |
| Horse | EHV-1 | 90c16 | AII80643.1 | KF644566.1 |
| Horse | EHV-1 | AB4 | AAT67287.1 | AY665713.1 |
| Horse | EHV-1 | FL06 | AII80724.1 | KF644567.1 |
| Horse | EHV-1 | HH1 | BAR43394.1 | AB992258.1 |
| Horse | EHV-1 | NMKT04 | AII80804.1 | KF644568.1 |
| Horse | EHV-1 | NY03 | AII80884.1 | KF644569.1 |
| Horse | EHV-1 | NY05 | AII80964.1 | KF644570.1 |
| Horse | EHV-1 | OH03 | AII81044.1 | KF644571.1 |
| Horse | EHV-1 | T953 | AKQ19073.1 | KM593996.1 |
| Horse | EHV-1 | V592 | AAS45914.1 | AY464052.1 |
| Horse | EHV-1 | VA02 | AII81124.1 | KF644572.1 |
| Gazelle | EHV-1 | 94-137 | AII81363.1 | KF644575.1 |
| Onager | EHV-1 | T529 10/84 | AII81761.1 | KF644580.1 |
| Zebra | EHV-1 | T616 | AII81283.1 | KF644574.1 |
| Horse | EHV-3 | AR/2007/C3A | AIL02947.1 | KM051845.1 |
| Horse | EHV-4 | NS80567 | AAC59546.1 | AF030027.1 |
| Donkey | EHV-8 | 804/87 | [AGZ94836.1](https://www.ncbi.nlm.nih.gov/protein/557362390) | /**^3^** |
| Horse | EHV-8 | Wh | AFI33165.1 | JQ343919.1 |
| Gazelle | EHV-9 | P19 | BAH02455.1 | AP010838.1 |
| Giraffe | EHV-9 | Neurological strain | /**^3^** | /**^3^** |
| Grevy’s zebra | EHV-9 | CG2 (lytic EHV-9) | AOS50781.1 | /**^3^** |
| Grevy’s zebra | EHV-9 | CG3 (lytic EHV-9) | AOS50782.1 | /**^3^** |
| Grevy’s zebra | EHV-9 | CG6 (latent EHV-9) | AOS50784.1 | /**^3^** |
| Plains zebra | EHV-9 | WP1 (lytic EHV-9) | AOS50780.1 | /**^3^** |

Abbreviations: EHV-1, equid herpesvirus 1; EHV-3, equid herpesvirus 3; EHV-4, equid herpesvirus 4; EHV-8, equid herpesvirus 8; EHV-9, equid herpesvirus 9.

^1^ Amino acid sequence. ^2^ Nucleotide sequence. ^3^ /, genome sequence not available
