# Supplementary material for: Equid herpesvirus 8: Complete genome sequence and association with abortion in mares
Source: PLoS One. 2018 Feb 7;13(2):e0192301. doi: 10.1371/journal.pone.0192301 (PMC5802896; doi:10.1371/journal.pone.0192301)
Supplement: S2 Table — (DOCX) [file pone.0192301.s002.docx]

**S2 Table. Number of amino acid sequence differences among the Irish EHV- 8 strains.** Comparison is made to EHV-8/IR/2003/19, and excludes tandem reiterations.

| **ORF** | **HSV-1**  **equivalent** | | **Protein** | **EHV- 8**  **/IR/2010/47** | **EHV- 8**  **/IR/2010/16** | **EHV- 8**  **/IR/2015/40** |
| --- | --- | --- | --- | --- | --- | --- |
| 2 | _ | | Membrane protein V1 | / | / | I179V***^1^** |
| 3 | _ | | Myristylated tegument protein CIRC | / | / | R227H* |
| 4 | UL55 | | Nuclear protein UL55 | / | / | / |
| 5 | UL54 | | Multifunctional expression regulator | S156A | / | V343I |
| 6 | UL53 | | Envelope glycoprotein K | / | / | N94S* |
| 7 | UL52 | | Helicase-primase primase subunit | N887T | / | A279V*, P704H* |
| 8 | UL51 | | Tegument protein UL51 | R13K, N216T | / | N216T |
| 9 | UL50 | | Deoxyuridine triphosphatase | T73A, T265M | / | T73A, S183I |
| 10 | UL49A | | Envelope glycoprotein N | / | / | S71T* |
| 12 | UL48 | | Transactivating tegument protein VP16 | / | / | Y435H* |
| 13 | UL47 | | Tegument protein VP13/14 | A714V | / | D150_E151insD, A165T*, H171Q* Y178D*, E196A* |
| 14 | UL46 | | Tegument protein VP11/12 | I331T | I331T | N15T*, I331T |
| 15 | UL45 | | Membrane protein UL45 | / | / | R57K* |
| 16 | UL44 | | Envelope glycoprotein C | / | / | T38A* |
| 18 | UL42 | | DNA polymerase processivity subunit | S376P | / | S376P |
| 19 | UL41 | | Tegument host shut-off protein | / | / | / |
| 20 | UL40 | | Ribonucleotide reductase subunit 2 | T144K, N240D | / | V121M*, T144K,  N240D |
| 21 | UL39 | | Ribonucleotide reductase subunit 1 | / | / | / |
| 22 | UL38 | | Capsid triplex subunit 1 | E394Q | / | / |
| 23 | UL37 | | Tegument protein UL37 | I684T, E995D | / | I684T, E995D |
| 24 **^2^** | UL36 | | Large tegument protein | L1340I, A1532E,  A538T, S3295A | / | L1340I |
| 25 | UL35 | | Small capsid protein | S57N | / | / |
| 26 | UL34 | | Nuclear egress membrane protein | D195N | / | / |
| 27 | UL33 | | DNA packaging protein UL33 | N107D | / | N107D |
| 28 | UL32 | | DNA packaging protein UL32 | K377R, L485I,  Y489C | / | / |
| 30 | UL30 | | DNA polymerase catalytic subunit | Y880S, M1102V | S411A | V751A*, M862L,  Y880S, T1084S,  M1102V |
| 31 | UL29 | | Single stranded DNA-binding protein | N186D, A290T | N186D | N186D |
| 32 | UL28 | | DNA packaging terminase subunit 2 | / | / | / |
| 33 | UL27 | | Envelope glycoprotein B | / | / | E106G |
| 34 | - | | Protein V32 | / | / | P63T |
| 35 | UL26 | | Capsid maturation protease | / | / | A107E |
| 36 | UL25 | | DNA packaging tegument protein UL25 | / | / | S249N, H487Q* |
| 37 | UL24 | | Nuclear protein UL24 | / | / | / |
| 41 | UL20 | | Envelope protein UL20 | / | / | / |
| 42 | UL19 | | Major capsid protein | S962A | / | A885S*, S962A |
| 44_47 | UL15 | | DNA packaging terminase subunit 1 | L145F | / | L145F, V180D,  V220I* |
| 45 | UL17 | | DNA packaging tegument protein UL17 | / | / | A275S* |
| 46 | UL16 | | Tegument protein UL16 | / | / | L218M* |
| 48 | UL14 | | Tegument protein UL14 | / | / | / |
| 49 | UL13 | | Tegument serine/threonine protein kinase | / | / | A220V |
| 54 | UL8 | | Helicase-primase subunit | E440Q | / | T446A, S688P* |
| 55 | UL7 | | Tegument protein UL7 | / | / | L13M* |
| 56 | UL6 | | Capsid portal protein | / | / | H506Q, S691Y* |
| 57 | UL5 | | Helicase-primase helicase subunit | / | / | S604N*, F755V* |
| 58 | UL4 | | Nuclear protein UL4 | A173P | / | A173P |
| 59 | - | | Protein V57 | / | / | H53R |
| 61 | UL2 | | Uracil-DNA glycosylase | Q109R, L132P | / | N82S*, Q109R |
| 63 | RL2 | | Ubiquitin E3 ligase ICP0 | S359G, P378S | / | D312E,  P373­_S374insS,  G456W* |
| 64 | RS2 | | Transcriptional regulator ICP4 | P69_S70insP, S75P,  P86A, T87S,  T87_T88insGPP,  T89P, A90P,  G91A, A93P,  L111M, A116V, A118P, R123Q, G129S, G130S, D131G, T132G, A133G, P135A,  G136S, G138D,  138_139insPVALDP306Q, G365A, T925A, A935V, H1426R | S1221N | Q149H, P150A, P153S, G159R, G160R, G161del, V164A, D183del, A184del, D185E,  V188A, T925A,  A935V, A1085S* |
| 65 | US1 | | Regulatory protein ICP22 | N39D | / | / |
| 66 | US10 | | Virion protein US10 | A228T | / | / |
| 68 | US2 | | Virion protein US2 | A83S, T274A | / | / |
| 69 | US3 | | Serine/threonine protein kinase | / | / | / |
| 70 | US4 | | Envelope glycoprotein G | R385K | / | / |
| 71**^2^** | US5 | | Envelope glycoprotein J | S502R,  T544_T545ins6  S600L, A827V | / | T544_T545ins6 |
| 72 | US6 | | Envelope glycoprotein D | V336I | / | / |
| 73 | US7 | | Envelope glycoprotein I | / | / | I255M |
| 74 | US8 | | Envelope glycoprotein E | T17I, A104E | A104E | A104E, A130T* |
| 75 | US8A | | Membrane protein US8A | E42A | / | N41S*, E42A |
|  | |  | Total Number of Differences | 87 | 5 | 86 |

Abbreviations: EHV-8, equid herpesvirus 8; HSV-1, herpes simplex virus type 1; ORF, open reading frame; UL, US, RL and RS, genes located in U_L_, U_S_, TR_L_/IR_L_, TR_S_/IR_S_, respectively (U_L_, unique long region; U_S_, unique short region; TR_L_/IR_L_ and TR_S_/IR_S,_ terminal and internal inverted repeats flanking U_L_ and U_S_, respectively); VP, viral protein; ICP, infected cell protein

* Unique to strain EHV- 8/IR/2015/40.

/ No amino acid difference(s).

^1^ Relative to EHV-8/IR/2003/19.

^2^ Tandem reiterations not included.

^3^ Remainder of ORF71 excluded from the analysis due to major sequence differences and truncation of coding region in EHV-8 strain Wh.
